# How We Sleep, How We Move, How Long We Expect to Live: An Integrative Review of Lifestyle Behaviors and Subjective Life Expectancy

**DOI:** 10.3390/nu18030515

**Published:** 2026-02-03

**Authors:** Oana Pătru, Andrei Păunescu, Andreea Bena, Silvia Luca, Cristina Văcărescu, Andreea-Iulia Ciornei, Mirela Virtosu, Bogdan Enache, Constantin-Tudor Luca, Simina Crisan

**Affiliations:** 1 Cardiology Department, “Victor Babes” University of Medicine and Pharmacy, 2 Eftimie Murgu Sq., 300041 Timisoara, Romania; oana.patru@umft.ro (O.P.); silvia.luca@umft.ro (S.L.); cristina.vacarescu@umft.ro (C.V.); bogdan.enache@umft.ro (B.E.); constantin.luca@umft.ro (C.-T.L.); simina.crisan@umft.ro (S.C.); 2 Research Center of the Institute of Cardiovascular Diseases Timisoara, 13A Gheorghe Adam Street, 300310 Timisoara, Romania; 3 Doctoral School, “Victor Babes” University of Medicine and Pharmacy, 2 Eftimie Murgu Sq., 300041 Timisoara, Romania; andreea.ciornei@umft.ro (A.-I.C.); daniela.cozma@umft.ro (M.V.); 4 Department of Urology, “Pius Brînzeu” Emergency Clinical County University Hospital, 300723 Timisoara, Romania; ai.paunescu@gmail.com; 5 Discipline of Endocrinology, Second Department of Internal Medicine, “Victor Babes” University of Medicine and Pharmacy, 2 Eftimie Murgu Sq., 300041 Timisoara, Romania; 6 Center for Molecular Research in Nephrology and Vascular Disease, “Victor Babes” University of Medicine and Pharmacy, 2 Eftimie Murgu Sq., 300041 Timisoara, Romania; 7 Institute of Cardiovascular Diseases Timisoara, 13A Gheorghe Adam Street, 300310 Timisoara, Romania

**Keywords:** sleep quality, circadian rhythm, physical activity, nutrition, subjective life expectancy, psychological aging, healthspan, longevity, health behaviors, integrative model

## Abstract

**Background**: Sleep quality (SQ) and physical activity (PA) are among the strongest behavioral determinants of healthy aging, while dietary behavior and psychological factors act as complementary modulators of these relationships. Although each domain has been studied extensively, their combined influence on subjective life expectancy (SLE)—an individual’s perceived likelihood of living to an advanced age—remains largely unexplored. **Methods**: This narrative review synthesizes evidence from sleep science, exercise physiology, behavioral medicine, and psychological aging. Literature published between January 2015 and 15 December 2025 was examined across PubMed, Scopus, and Web of Science using integrative keyword strategies. Studies addressing SQ, PA, circadian rhythms, psychological health, SLE, or aging-related outcomes were included. **Results**: The review identifies several converging pathways linking sleep and PA to aging trajectories. Sleep architecture, circadian stability, metabolic regulation, inflammatory balance, and autonomic function represent key biological mechanisms. PA contributes through improvements in mitochondrial efficiency, VO_2_max, muscle metabolism, and anti-inflammatory signaling (IL-6 as a myokine). Across studies, both sleep and PA strongly influence psychological health, health perception, and future-oriented expectations, within a broader lifestyle context supported by nutritional status and dietary quality. SLE emerges as a central psychological mediator that shapes motivation, adherence to health behaviors, and long-term health outcomes. Contextual moderators—including age, gender, socioeconomic status, cultural norms, and wearable technology engagement—further influence these relationships. **Conclusions**: SQ and PA form the core behavioral components of a dynamic system that is further shaped by dietary behavior and psychological well-being and centered on SLE. Our proposed integrative model positions SLE as a key psychological link between lifestyle behaviors and longevity. This framework is hypothesis-generating and requires empirical validation through future longitudinal and interventional studies, underscoring the need for multidomain research integrating behavioral, biological, nutritional and psychological indicators of aging.

## 1. Introduction

Longevity and healthy aging have become central priorities in modern preventive medicine, driven by demographic transitions and the rising global burden of chronic diseases. In the context of healthy aging, lifestyle behaviors such as PA and SQ do not operate in isolation but form an interdependent behavioral system that is strongly influenced by dietary patterns and nutritional status. Sleep and PA jointly influence circadian regulation, metabolic homeostasis, inflammatory balance and psychological well-being—core processes underlying both biological aging and subjective perceptions of longevity—while diet and nutritional status modulate these processes. Although genetic factors contribute to lifespan, converging evidence indicates that modifiable lifestyle behaviors—including SQ, PA and dietary habits—play a decisive role in shaping both healthspan and lifespan. Sleep duration, sleep continuity, and circadian rhythm stability have been consistently associated with cardiometabolic health, cognitive functioning, emotional regulation, and mortality risk [1,2,3]. Likewise, regular PA and reduced sedentary behavior exert profound benefits across cardiovascular, metabolic, musculoskeletal, and neurocognitive systems, while also reducing premature mortality. These behavioral determinants are further linked to biological aging processes, including systemic inflammation, oxidative stress, mitochondrial function, and telomere dynamics, making them central components of contemporary healthy aging models [4,5,6].

Alongside these physiological pathways, a psychological construct known as SLE—an individual’s personal estimation of how long they expect to live—has emerged as an increasingly meaningful predictor of health trajectories [7]. Longitudinal studies show that lower SLE is associated with reduced adherence to preventive behaviors, poorer self-rated health, increased morbidity, and higher mortality, independent of traditional clinical risk factors. Conversely, individuals who perceive a longer future lifespan tend to engage more consistently in health-promoting behaviors such as regular exercise, structured sleep routines, and reduced sedentary time. SLE therefore functions not only as a cognitive appraisal of aging prospects but also as a motivational determinant of lifestyle behaviors [8,9].

Despite the rapidly growing body of work on sleep, PA, and behavioral determinants of aging, the existing literature remains fragmented. Research typically examines these domains in isolation: PA interventions and lifestyle programs assessing objective or subjective sleep outcomes in older adults; analyses of how sleep duration or SQ modulate the effects of exercise on cognitive or functional performance; umbrella reviews of non-pharmacological interventions targeting sleep disorders; or cohort studies linking PA, sedentary time, diet, or sleep to biological aging indicators and mortality [10]. Although dietary behavior is frequently considered alongside other lifestyle factors, it is rarely integrated into conceptual models that simultaneously examine sleep, PA, and psychological constructs. Likewise, several studies have explored lifestyle factors in relation to subjective health or quality of life (QoL). However, none of these investigations incorporate SLE, nor do they examine how sleep and PA jointly influence an individual’s perception of their own longevity. The psychological dimension of aging—how long one expects to live—remains largely absent from integrative lifestyle frameworks, despite its demonstrated importance for long-term health behavior and mortality risk [11,12].

This omission represents a significant conceptual gap. The absence of SLE from existing models prevents a full understanding of the bidirectional feedback loops through which SQ and PA shape, and are shaped by psychological expectations of longevity. These interactions have profound implications: sleep and PA influence biological aging and health perception; SLE influences motivation, behavior maintenance, and adherence; and behavior change influences both physiological and psychological aging markers. Without an integrative framework, these relationships remain compartmentalized, limiting opportunities for designing comprehensive preventive interventions and for advancing theoretical perspectives on healthy aging.

The present narrative review addresses this gap by offering the first review to integrate SLE into existing SQ and PA frameworks, extending them by positioning SLE as a central psychological mediator linking lifestyle behaviors to perceived longevity. While dietary behavior and nutritional status are not examined as primary analytical domains, they are conceptually positioned as important contextual and biological modulators that interact with the same metabolic, inflammatory, and psychological pathways discussed in this framework. Our work brings together evidence from epidemiology, sleep and exercise science, nutrition, psychology, and gerontology to examine how lifestyle behaviors influence not only biological aging and health outcomes but also perceptions of longevity, and how these perceptions may in turn shape future behaviors. We propose a multidimensional conceptual framework through which sleep patterns, movement behaviors, and longevity expectations interact; highlight shared mechanisms—including circadian regulation, inflammatory and metabolic pathways, executive function, emotional well-being, and self-regulatory capacity; and identify research gaps that future observational and interventional studies should address.

By situating SLE at the intersection of lifestyle behaviors and psychological aging, this review offers a novel perspective with direct implications for preventive medicine, behavioral counseling, public health strategy, and personalized lifestyle interventions. Our aim is to contribute a comprehensive, integrative understanding of how individuals sleep, how they move, and ultimately, how long they expect to live.

## 2. Materials and Methods

This narrative review was developed to synthesize and integrate evidence across sleep science, PA research, behavioral medicine, and psychological aging, with a particular emphasis on SLE. In this manuscript, PA is used as an umbrella term referring to any bodily movement that results in energy expenditure; exercise denotes a structured and planned subtype of PA, while movement behavior refers to the full 24 h spectrum including PA, sedentary behavior, and sleep. A focused, theory-driven search strategy was employed to ensure relevance to the review’s central question and to avoid the overly broad retrieval typical of lifestyle-related terms. Literature searches were conducted in PubMed, Scopus, Web of Science, and Google Scholar for articles published between January 2015 and 15 December 2025. To capture studies at the intersection of the domains of interest, the search strategy prioritized combined keywords linking sleep and longevity (e.g., “sleep quality AND life expectancy”), physical activity and aging (e.g., “physical activity AND mortality,” “sedentary behavior AND healthspan”), and lifestyle behaviors with subjective or perceived longevity (e.g., “subjective life expectancy,” “perceived longevity,” “self-rated health AND aging”). Broad single-domain terms were intentionally avoided as primary search strings in order to reduce non-specific results.

The initial combined search yielded 1482 records (PubMed: 612; Scopus: 544; Web of Science: 326). Titles and abstracts were screened for conceptual relevance. Duplicates (*n* = 412) were removed, leaving 1070 unique records. Of these, 124 articles were retained for full-text relevance screening. Inclusion criteria were: peer-reviewed articles published between January 2015 and up to 15 December 2025; adult populations (≥18 years); studies addressing at least one of the following domains: sleep (architecture, quality, duration, circadian biology), PA or movement behavior, psychological health or QoL, biological or functional indicators of aging; review articles, meta-analyses, cohort studies, randomized trials, or mechanistic reviews. Exclusion criteria were: studies on pediatric populations; manuscripts without clear relevance to at least one core domain; purely technical or methodological sleep studies (e.g., algorithm validation); non-English publications. After applying these criteria, 41 studies remained eligible for qualitative synthesis. Because the purpose of the review was to construct an integrative conceptual model, a subset of 10 studies was purposively selected based on explicit operational criteria: coverage of at least two core domains of the model (sleep, PA, and/or physiological or psychological aging-related outcomes); use of robust study designs, including systematic reviews, large population-based cohort studies, or mechanistic narrative reviews; publication in high-impact, peer-reviewed journals between January 2015 and 15 December 2025; clear relevance for mapping biological and/or psychological mechanisms underlying healthy aging; and methodological transparency in exposure and outcome assessment. Although no formal risk-of-bias tool was applied, priority was given to studies characterized by large sample sizes, longitudinal designs where applicable, validated measurement instruments, and consistency in outcome reporting. Articles were eligible if they were peer-reviewed, published in English, involved adult populations, and examined relationships between sleep, PA or sedentary behavior, biological or functional aging markers, subjective health perceptions, or SLE. Cohort studies, cross-sectional analyses, interventional trials, systematic reviews, and meta-analyses were all eligible for inclusion. Studies that lacked conceptual or empirical relevance to at least one of the review’s behavioral or psychological domains were excluded, as were case reports, conference abstracts without accessible full text, pediatric studies, and non-peer-reviewed sources. Because SLE is rarely assessed in lifestyle literature, studies that did not explicitly measure SLE were retained when they provided mechanistic or theoretical insights essential for developing an integrative conceptual model.

Data extraction followed an iterative, interpretive process consistent with established narrative review methodology. Studies were examined for methodological rigor, population characteristics, measurement quality, type of aging-related outcomes, and relevance to psychological constructs. No formal risk-of-bias tool or quantitative synthesis was applied, as the goal was not to pool effect sizes but to integrate heterogeneous findings into a coherent conceptual framework. Priority was given to high-quality cohort studies, meta-analyses, systematic reviews, and well-designed interventional research. Divergent findings were compared and interpreted to identify consistent mechanisms and cross-domain interactions, with thematic saturation used to determine adequacy of the evidence base.

The review was conducted by a multidisciplinary team spanning various medical specialties, psychology, and clinical pharmacy. This diverse expertise ensured a comprehensive interpretation of the biological, behavioral, and psychological evidence underpinning the relationships among sleep, PA, and SLE. A narrative design was deemed most appropriate because the question addressed requires conceptual integration across fields that are rarely studied together. The heterogeneity of outcomes, methodologies, and theoretical frameworks precludes meaningful meta-analytic pooling; instead, narrative synthesis enables the development of a multidimensional model and the identification of research gaps across the interconnected domains of healthy and perceived aging.

**Figure 1:**
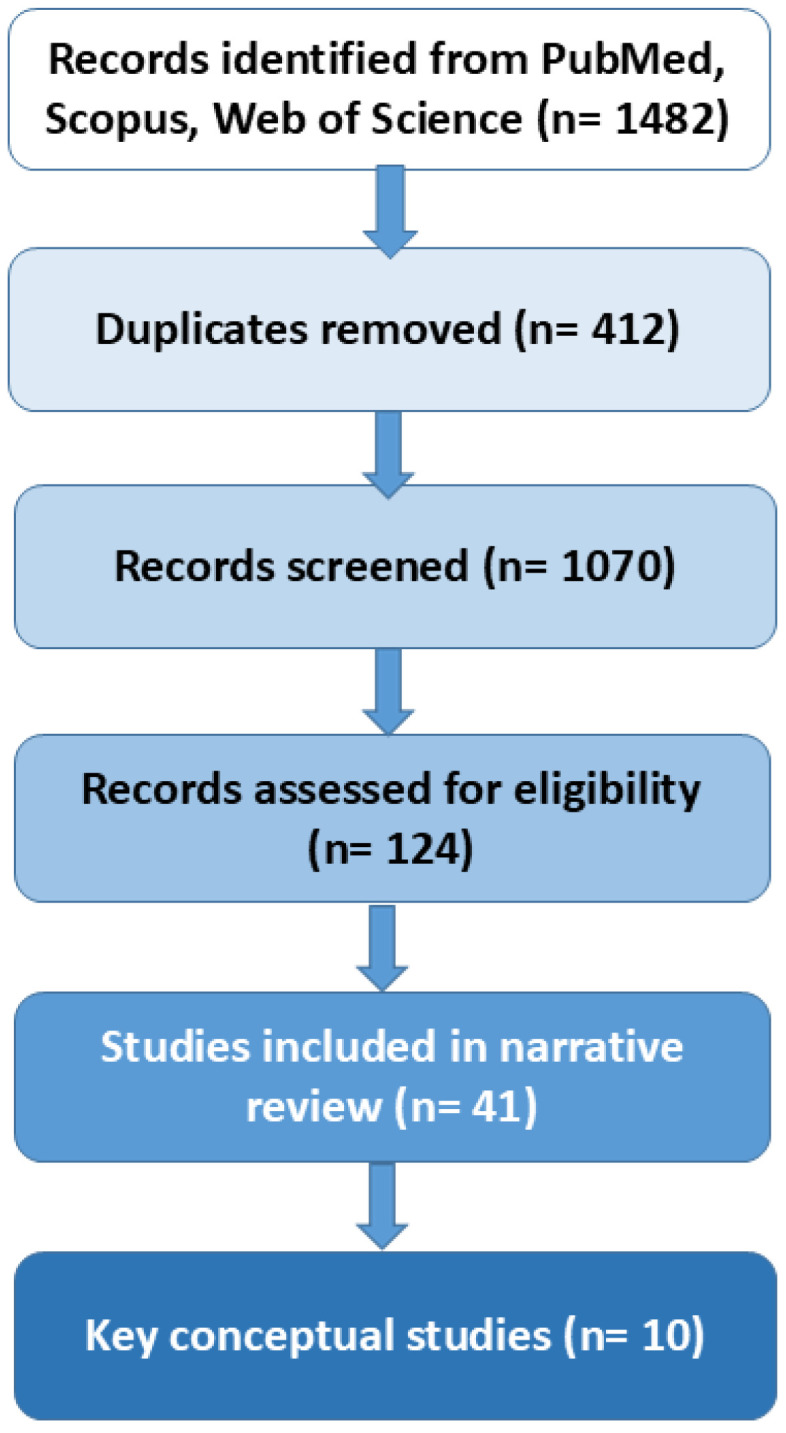
Narrative review flow diagram summarizing study identification, screening, and inclusion.

A simplified flow diagram summarizing record identification (*n* = 1482), screening (*n* = 1070), full-text assessment (*n* = 124), and final inclusion (*n* = 41; with 10 highlighted as key conceptual studies) is provided in Figure 1.

## 3. Results

To synthesize the heterogeneous evidence across sleep, PA, autonomic regulation, and aging-related outcomes, we conducted a narrative integration of all eligible studies identified through the literature search. To facilitate conceptual clarity, we mapped ten representative peer-reviewed studies in Table 1 as illustrative examples of the core domains underpinning our conceptual model. These exemplar studies include systematic reviews, observational analyses, and mechanistic papers published between January 2015 and 15 December 2025. While Table 1 highlights selected studies for illustrative and integrative purposes, the Results section draws on the full body of included literature to examine converging biological, behavioral, and psychological pathways linking SQ, PA, and healthy aging. Together, this broader synthesis illustrates the multidimensional and interdependent nature of lifestyle and psychological determinants of longevity.

**Figure 2:**
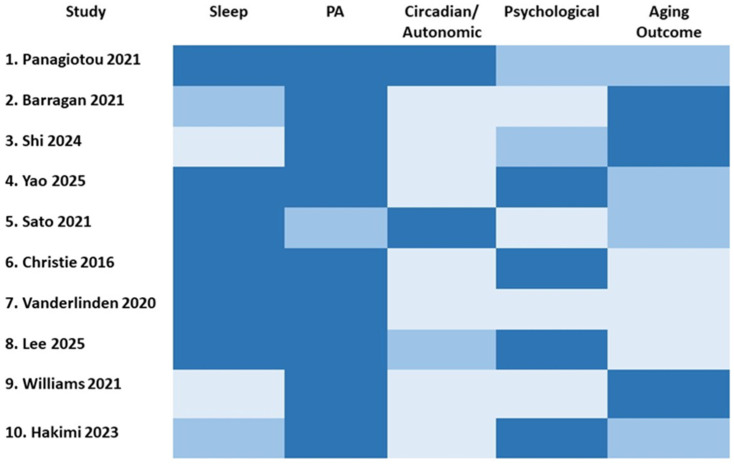
Heatmap summarizing the coverage of key domains across the ten studies included in the narrative synthesis. Darker shades indicate stronger emphasis within each study. The domains reflect core components of the integrative model: sleep, PA, circadian/autonomic mechanisms, psychological processes, and aging-related outcomes. The figure illustrates heterogeneity in the literature and highlights complementary contributions across mechanistic, behavioral, and population-based approaches [10,11,13,14,15,16,17,18,19,20].

To visualize the multidimensional coverage of domains across the included studies, we constructed a heatmap synthesizing the extent to which each study addressed sleep, PA, circadian/autonomic mechanisms, psychological factors, and aging outcomes as seen in Figure 2.

### 3.1. Sleep and Healthy Aging

A substantial body of evidence demonstrates that sleep is a fundamental determinant of both healthspan and longevity. While early research focused primarily on sleep duration, contemporary studies highlight the indispensable role of SQ, sleep architecture, and circadian rhythm stability. Sleep architecture undergoes predictable changes across the lifespan, with reductions in slow-wave sleep (SWS), more fragmented sleep, and alterations in REM expression. These changes are not merely epiphenomena of aging but contribute actively to its progression. SWS is intimately involved in metabolic regulation, synaptic downscaling, memory consolidation, growth hormone release, and cellular repair; reductions in SWS have been associated with impaired glucose tolerance, elevated inflammatory cytokines, and accelerated cognitive decline. REM sleep, equally crucial, supports emotional regulation, learning, and autonomic balance. Disruptions in either domain correlate with poorer cardiometabolic outcomes and diminished physiological resilience [13,17,21,22,23].

SQ exerts profound effects on biological aging markers. Several studies link insomnia symptoms, irregular sleep–wake rhythms, and sleep fragmentation with shorter leukocyte telomere length, one of the most robust indicators of cellular aging. Mechanistically, insufficient or nonrestorative sleep increases oxidative stress, reduces antioxidant defenses, activates the sympathetic nervous system, and elevates systemic inflammatory markers—all pathways known to accelerate telomere attrition. HRV, a widely used index of autonomic function, is strongly modulated by SQ. Low HRV reflects heightened sympathetic activation and decreased parasympathetic tone, and consistently predicts increased mortality risk, metabolic dysfunction, and diminished stress resilience. Individuals with poor sleep exhibit chronically lower HRV, suggesting autonomic dysregulation as a key mediator connecting sleep to aging processes [14,24,25,26,27,28].

The relationship between sleep and PA provides an additional pathway linking sleep to longevity. Experimental and observational research shows that poor sleep impairs next-day energy, reduces willingness to engage in PA, increases perceived exertion, and disrupts appetite-regulating hormones such as leptin and ghrelin. These changes promote sedentary behavior, weight gain, and increased cardiometabolic risk. Conversely, regular PA improves sleep continuity, increases slow-wave sleep, and stabilizes circadian rhythms, although these effects vary by age, fitness level, and type of exercise. Thus, sleep and PA operate as mutually reinforcing—or mutually deteriorating—components of behavioral health [10,13,14,15,29,30,31,32].

Sleep may also influence subjective dimensions of aging. Individuals reporting higher SQ consistently demonstrate better self-rated health, greater vitality, more stable mood, and a more optimistic outlook on future aging. These perceptions translate into higher SLE, suggesting that sleep shapes not only physiological aging trajectories but also psychological expectations regarding future lifespan. In this sense, sleep emerges as a determinant of both objective and perceived longevity [18,20,33,34,35,36,37,38,39].

### 3.2. PA, Sedentary Behavior and Longevity

PA is among the strongest modifiable predictors of longevity, with research consistently demonstrating robust dose–response relationships between activity levels and mortality risk. Even small increases in activity yield substantial reductions in morbidity, particularly when individuals transition from sedentary to moderately active lifestyles. Different modalities of PA confer unique anti-aging benefits. Aerobic exercise improves endothelial function, mitochondrial efficiency, insulin sensitivity, and cardiopulmonary fitness, with cardiorespiratory capacity—measured as VO_2_max—emerging as one of the strongest independent predictors of mortality known in clinical science. Resistance training counters sarcopenia, preserves bone density, enhances metabolic health, and reduces frailty, thereby supporting physical independence in older age. High-intensity interval training (HIIT), despite its brief duration, induces powerful metabolic and mitochondrial adaptations and is increasingly recognized as a potent anti-aging stimulus even in older adults. Comprehensive exercise programs combining aerobic and resistance training produce the most consistent improvements across domains [15,19,40,41,42,43,44,45].

PA is likely to influence aging not only through systemic physiological benefits but also via molecular and cellular pathways. Muscle contractions stimulate the release of IL-6 as a myokine, which—distinct from its pro-inflammatory role in immune contexts—acts as a potent anti-inflammatory signal during exercise, promoting the release of IL-10 and inhibiting TNF-α. This shift toward an anti-inflammatory milieu counters the low-grade inflammation characteristic of aging (“inflammaging”). Regular exercise also enhances mitochondrial biogenesis, supports autophagy, and reduces oxidative stress, thereby decelerating cellular aging mechanisms [19,46,47,48,49,50,51].

In contrast, sedentary behavior may exert detrimental effects independent of PA levels. Adults who meet recommended activity guidelines but spend prolonged periods sitting still exhibit higher mortality, impaired endothelial function, diminished HRV, and faster telomere shortening. Isotemporal substitution analyses reveal that even replacing 20–30 min of daily sedentary time with light activity reduces markers of biological aging, highlighting the importance of PA throughout the day rather than exercise alone [16,20,52,53,54].

PA also plays a crucial psychological role in aging. Regular exercise enhances mood, increases self-efficacy, improves cognitive functioning, and strengthens the sense of personal control over health trajectories. These psychological benefits contribute significantly to subjective assessments of vitality and well-being, which in turn influence perceived longevity. Individuals who maintain regular PA tend to describe themselves as healthier, more resilient, and more capable of aging successfully, and consequently report higher subjective life expectancy. This positions PA as a behavioral determinant of both biological aging and psychological future orientation [15,16,55,56,57].

### 3.3. SLE and Psychological Dimensions of Aging

SLE represents an individual’s beliefs about how long they expect to live and serves as a powerful psychological indicator of aging. SLE is highly predictive of mortality, even after adjusting for clinical diagnoses, functional impairments, sociodemographic variables, and health behaviors. Individuals with low SLE exhibit higher mortality risk, often due to lower engagement in preventive behaviors, poorer mental health, and diminished motivation for long-term lifestyle investments. Conversely, individuals reporting higher SLE tend to engage more consistently in exercise, maintain healthier diets, participate in social activities, and adhere to medical treatments [15,57].

SLE is shaped by multiple factors, including self-rated health, functional capacity, emotional well-being, optimism, sense of control, coping efficacy and lifestyle-related influences such as dietary quality and nutritional status. People who feel energetic, emotionally resilient, and physically capable project their lives further into the future. Conversely, chronic stress, depressive symptoms, insomnia, fatigue, and sedentary lifestyle predict lower perceived longevity. SLE also mirrors an individual’s perceived trajectory of aging: those who anticipate decline or disability often adjust their behavioral investments downward, creating a cycle in which expectations influence behaviors and behaviors reinforce expectations [17,20,58,59,60].

Importantly, although sleep and PA influence subjective health, vitality, and optimism, no studies have evaluated their combined effect on SLE. Conceptually, sleep and PA contribute to SLE through intertwined biological and psychological pathways—by improving energy, mood, cognitive clarity, physiological resilience, and perceived personal control. Yet these relationships have not been systematically integrated, leaving an important conceptual gap in lifestyle-based models of aging [61,62,63].

### 3.4. Integrative Perspective Across Domains

To our knowledge, no studies in the medical literature have directly examined the separate effects of changes in sleep patterns or PA on SLE as a primary outcome. The converging findings from these three domains suggest that sleep, PA, and SLE are part of a dynamic, mutually reinforcing system that shapes both objective and perceived aging trajectories. High-quality sleep improves daytime functioning, emotional balance, and perceived health. These improvements, in turn, support higher SLE by enhancing optimism, confidence, and the sense that one’s future is worth investing in. Higher SLE fosters motivation for healthy behaviors, reinforcing engagement in regular PA and protective lifestyle routines. These routines often co-occur with healthier dietary patterns that further support metabolic and psychological resilience. PA further enhances SQ and circadian alignment, improves metabolic and inflammatory profiles, and strengthens psychological resilience—closing the loop in a virtuous cycle of healthy aging [59,60,61].

**Figure 3:**
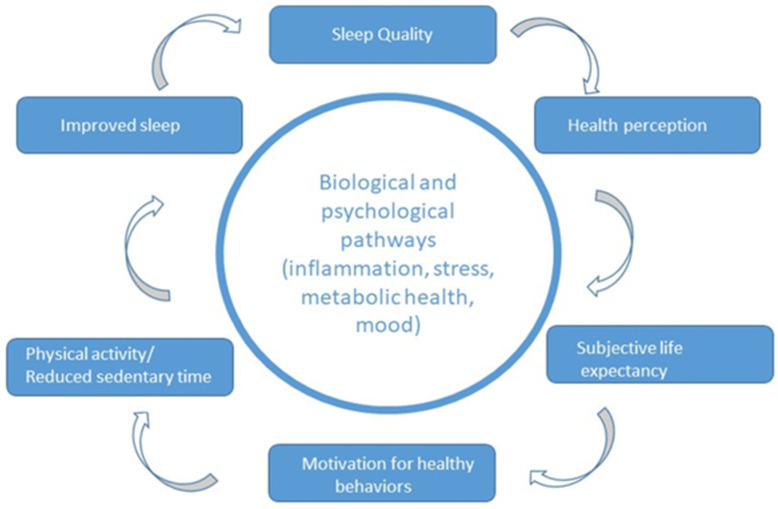
Virtuous and vicious feedback loops linking SQ, health perception, SLE, motivation, and PA.

The inverse is equally plausible, the vicious loop: sleep disruptions lead to lower vitality and poorer health perception, reducing SLE and weakening motivation to engage in PA. Inactivity then exacerbates sleep problems, accelerating physiological and psychological decline in a self-perpetuating negative cycle. This bidirectional, cyclical system underscores the need for integrative models of aging that address behavioral, biological, and psychological dimensions simultaneously as seen in Figure 3 [27,29,30].

## 4. Discussion

**Figure 4:**
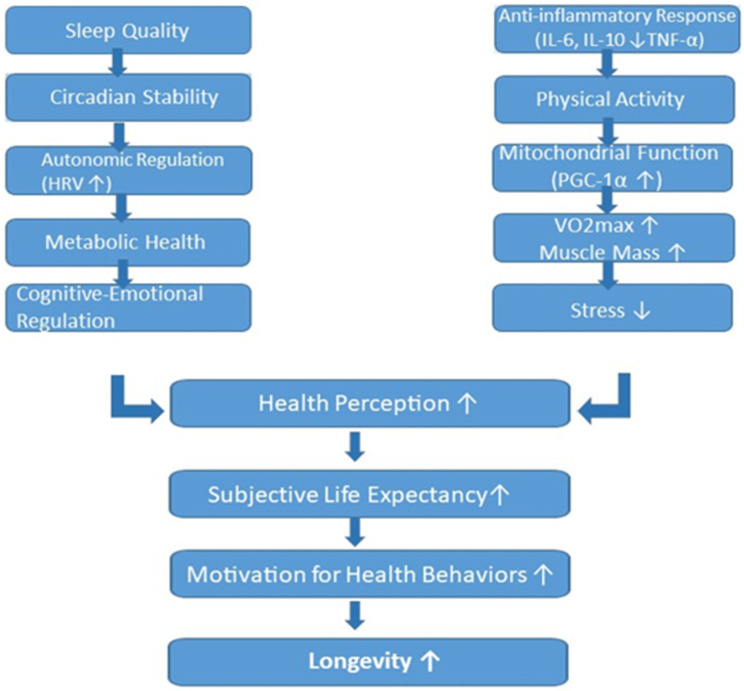
Mechanistic pathways linking SQ, PA and broader lifestyle factors to health perception, SLE, and longevity. Arrows indicate the direction of change: ↑ increase, ↓ decrease.

The present narrative review synthesizes evidence across sleep science, PA research, and psychological aging to articulate a unified perspective on how lifestyle behaviors, including sleep, PA and dietary patterns, as complementary lifestyle components, shape both objective and subjective aging trajectories. An overview of the biological and psychological pathways linking sleep and PA to SLE is presented in Figure 4.

Although each of these domains has been studied extensively, their interconnections reveal a more complex and dynamic model of healthy aging than previously recognized. In particular, the integration of SLE into lifestyle-based frameworks offers a novel and theoretically rich understanding of how individuals perceive, regulate, and enact their long-term health behaviors.

SQ emerged as a central determinant of aging outcomes, influencing metabolic regulation, inflammatory pathways, cognitive performance, emotional balance, and autonomic function. PA similarly contributes to biological resilience, cardiometabolic health, mitochondrial efficiency, anti-inflammatory processes, and psychological vitality. The literature reviewed demonstrates that these two systems—sleep and PA—are inherently interdependent. Improvements in sleep tend to increase daily energy, motivation, and overall capacity for PA, while regular exercise supports healthier sleep architecture and stronger circadian alignment. These reciprocal influences create a behavioral ecology in which lifestyle choices, including sleep, PA and dietary habits, continuously reinforce or erode one another.

What has been largely overlooked in prior research is the psychological bridge between these behaviors: SLE, a construct that captures how individuals envision their future lifespan. SLE is not merely a cognitive estimate but a motivational framework that shapes how much effort individuals invest in long-term health. Higher SLE may be associated with more favorable health behaviors, including greater engagement in regular PA, healthier sleep routines, more consistent adherence to balanced dietary behaviors, greater optimism, and enhanced self-regulation, whereas lower SLE may predict disengagement, reduced preventive care, sedentary lifestyles, less healthy dietary behaviors and higher mortality risk. The evidence suggests that sleep and PA may influence SLE by modulating health perception, vitality, emotional balance, and sense of personal control—variables that contribute to how individuals interpret their aging trajectory. This conceptual bridge provides new insight into why lifestyle interventions often succeed or fail depending on psychological readiness for long-term investment.

SLE is a valuable yet methodologically heterogeneous construct, with substantial variability across studies in both format and interpretability. Large population cohorts such as Health and Retirement Study (HRS) [64], the English Longitudinal Study of Ageing (ELSA) [65], the Survey of Health, Ageing and Retirement in Europe (SHARE) [66], and the Japanese Study of Ageing and Retirement (JSTAR) [67] typically assess SLE through probabilistic estimates (e.g., the likelihood of surviving to age 75). These measures show strong predictive validity for mortality and are particularly suitable for epidemiological modeling; however, they impose higher cognitive and numeracy demands, may be less intuitive for participants, and are less easily translated into clinical or behavioral counseling contexts. In contrast, other studies rely on numeric age predictions (e.g., “How old do you expect to become?”), which are simpler, more intuitive, and more easily applicable in patient-centered communication and behavioral interventions. Nevertheless, numeric formats are more susceptible to anchoring effects, cultural norms regarding lifespan, and transient changes in mood or health perception. These approaches therefore differ not only in cognitive demand and susceptibility to bias, but also in their interpretive and practical implications. Probabilistic measures are better suited for population-level risk estimation and longitudinal prediction, whereas numeric estimates may be more useful for motivating lifestyle change and facilitating discussions about future-oriented health behaviors. Furthermore, SLE is influenced by personality traits (e.g., optimism), socioeconomic conditions, and current physical and mental health status, which complicates causal inference across study designs. The absence of standardized measurement protocols limits direct comparability and may partially explain inconsistencies in reported associations with mortality and health behaviors. Future research would benefit from harmonized SLE assessment strategies and from integrating SLE with complementary constructs such as subjective age, future time perspective, and perceived health trajectories.

### 4.1. Integrative Model: A New Contribution to the Literature

Drawing from the reviewed evidence, we propose an integrative conceptual model in which SQ and PA represent the core behavioral drivers of the system, with SLE occupying a central psychological position linking lifestyle behaviors to motivation and long-term health trajectories. Dietary behaviors and nutritional status are included as transversal biological modulators that influence the same pathways without constituting primary analytical domains of the model. Sleep influences circadian rhythms, energy regulation, and emotional well-being, which affect both PA and psychological functioning. Psychological health—including optimism, self-efficacy, and coping ability—modulates SLE, which in turn shapes motivation for engaging in health-promoting behaviors. These behaviors, especially regular PA and structured routines, feed back into improved SQ and circadian alignment. Ultimately, the interplay between these components contributes to both biological longevity and perceived longevity.

**Figure 5:**
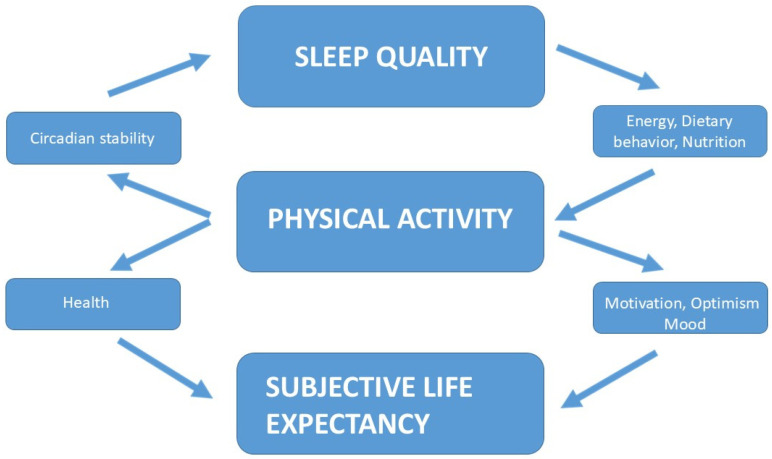
Conceptual integrative model linking SQ, PA, SLE and broader lifestyle behaviors.

The proposed model offers a unifying framework that reconciles behavioral, biological, and psychological determinants of aging. It emphasizes that longevity is not solely the product of physiological processes but also of individuals’ beliefs about their future and the motivational dynamics arising from those beliefs. By situating SLE within this behavioral—biological loop, our model introduces a mechanism that may explain individual differences in adherence to lifestyle interventions and the divergent aging trajectories observed across populations, as depicted in Figure 5.

Although nutrition is not examined as a primary analytical domain in the present review, nutritional status represents a clinically measurable and biologically meaningful modifier of aging trajectories. Recent evidence shows that parameters used to identify malnutrition in older adults are robust, practical, and closely linked to functional decline, inflammatory burden, and metabolic dysregulation [68]. These mechanisms overlap substantially with the biological pathways through which SQ and PA influence both biological and perceived aging. From this perspective, nutrition functions as a transversal biological amplifier or attenuator of the proposed sleep-PA-SLE system, rather than as a parallel independent pillar of the model.

To increase the empirical testability of the proposed model, several hypotheses can be derived for future longitudinal and interventional research. First, SLE is expected to mediate the relationship between lifestyle behaviors and long-term health outcomes, such that improvements in SQ and PA will be associated with higher SLE, which in turn will predict greater adherence to health-promoting behaviors and more favorable aging trajectories. Second, SES is expected to moderate these associations, with weaker effects in populations exposed to higher chronic stress and reduced access to health-supportive environments. Third, discrepancies between objective and subjective SQ are hypothesized to differentially influence SLE, such that individuals who perceive their sleep as poor despite objectively adequate sleep may exhibit lower SLE and reduced motivation for behavioral engagement. Fourth, the use of wearable devices is expected to strengthen the sleep–PA–SLE feedback loop by increasing self-monitoring, perceived control, and behavioral reinforcement. These hypotheses provide a framework for longitudinal, mediation, and moderation analyses that can empirically validate the proposed conceptual pathways.

The proposed framework is theoretical and hypothesis-generating, and it requires empirical validation through future longitudinal, experimental, and multivariate studies before causal inferences can be drawn.

### 4.2. Special Considerations

#### 4.2.1. Age

Aging modifies the interaction between sleep, PA, and psychological variables. Older adults exhibit more fragmented sleep, decreased slow-wave sleep, reduced circadian amplitude, and altered exercise responsiveness [10,20,34]. Also, age-related changes in appetite, nutrient absorption, and dietary routines may further interact with sleep and PA patterns [69]. These changes modify how lifestyle patterns translate into perceived health and longevity expectations. However, older populations also demonstrate higher sensitivity to lifestyle interventions, making this group particularly relevant for applying the integrative model.

#### 4.2.2. Gender

Gender differences exist in SQ, insomnia prevalence, PA patterns, and health perceptions. Women often report lower SQ and greater perceived stress [70], whereas men may engage in less preventive healthcare but have higher baseline SLE [71]. These factors may influence engagement with lifestyle interventions and the strength of the sleep-activity-SLE feedback loop.

#### 4.2.3. Socioeconomic Status (SES)

SES shapes lifestyle opportunities, stress exposure, health literacy, and perceptions of aging. Individuals with lower SES often experience circadian disruption due to shift work, limited access to exercise environments, food insecurity, poorer dietary quality and higher daily stress-factors that can diminish both SQ and SLE [72]. Understanding SES moderating effects is essential for equitable interventions.

#### 4.2.4. Cultural Differences

Cultural beliefs about aging, death, optimism, and long-term planning influence SLE. Some cultures value extended future orientation, while others accept shorter perceived lifespan as normative [73,74]. These cultural differences may modify how sleep and PA influence psychological expectations of longevity and should be considered when developing global aging interventions.

#### 4.2.5. Wearables and Digital Health as Behavioral Reinforcers

Wearable devices (actigraphy, fitness trackers, sleep analyzers) increasingly shape behavior by providing real-time feedback, accountability, and positive reinforcement. These tools may improve sleep hygiene, increase PA, and strengthen self-regulation [75]. Beyond lifestyle-focused research, parallel advances in clinical medicine continue to refine the assessment and management of age-related cardiovascular conditions in adult populations, underscoring the broader biomedical context in which healthy aging unfolds [76]. Importantly, they can also influence SLE by making progress visible and helping individuals perceive aging as modifiable rather than predetermined.

### 4.3. Limitations

This review has several limitations inherent to its narrative design. The search strategy, although structured and transparent, was not exhaustive and may have missed relevant studies outside the primary databases or the selected time window. The inclusion of heterogeneous study designs-ranging from mechanistic reviews to large cohort analyses—precludes direct comparison across methodologies. Because SLE is seldom examined together with sleep and PA, the conceptual synthesis relied partly on extrapolation from adjacent psychological constructs. Additionally, the selection of ten key studies for tabulation was purposeful rather than systematic, prioritizing conceptual relevance over representativeness. Finally, causal relationships among sleep, PA, psychological processes, and aging outcomes cannot be inferred from the current evidence base. These limitations highlight the need for more integrated and longitudinal research.

### 4.4. Gaps in Literature, Future Directions and Empirical Testing of the Model

Although evidence linking sleep, PA, and SLE is compelling, several important gaps remain. First, interventions explicitly targeting SLE are almost nonexistent. Given that SLE predicts mortality and influences lifestyle choices, future research should examine whether modifying SLE—through cognitive reframing, behavioral activation, or personalized health projections—can enhance engagement with sleep- and PA-focused interventions.

Second, greater standardization of SQ assessment is urgently needed. The use of heterogeneous tools (e.g., PSQI, actigraphy, polysomnography, self-report) limits comparability across studies and may obscure key mechanistic pathways; consensus approaches integrating both subjective and objective measures would strengthen the field.

Third, future research should integrate behavioral aging predictors with biological aging biomarkers, including telomere length, epigenetic clocks, inflammatory cytokines, heart rate variability, and mitochondrial markers. Such multidimensional designs would help clarify which pathways most strongly mediate relationships between lifestyle behaviors and SLE.

Fourth, existing evidence is geographically and demographically limited. Large, multicultural and cross-national studies are needed to understand how cultural norms, environmental exposures, socioeconomic gradients, and healthcare systems shape SLE and its interactions with sleep and PA.

Fifth, the directionality of these relationships remains unclear. Sleep, PA, and SLE likely influence one another in dynamic ways that vary across individuals and contexts; longitudinal and experimental designs, including mediation and time-series analyses, are required to disentangle these temporal pathways.

Future interventions should adopt integrative lifestyle frameworks rather than isolated behavior change strategies. Programs simultaneously targeting sleep hygiene, circadian stability, PA, dietary quality and psychological resilience may generate synergistic effects by activating the virtuous cycle proposed in our model. Clinically, assessing SQ, activity patterns, and longevity expectations as interconnected domains may improve identification of individuals at risk for poor adherence or declining health trajectories. Brief discussions about future health expectations in primary and geriatric care may further enhance motivation for lifestyle change, while public health strategies promoting 24-h movement guidelines, reduced sedentary behavior, and improved sleep literacy could support both objective and perceived longevity.

The proposed integrative model is amenable to empirical testing using longitudinal and multivariate analytic frameworks. Structural equation modeling and cross-lagged panel designs could examine bidirectional associations among sleep, PA, psychological variables, and SLE over time, while mediation analyses may clarify whether SLE functions as a psychological intermediary linking lifestyle behaviors to long-term outcomes. Incorporating objective behavioral measures alongside biological aging markers would enable multimodal validation of the proposed pathways, advancing the model toward clinical and public health applicability.

From a clinical perspective, SLE can be assessed using a brief and intuitive question such as: “How old do you realistically expect to become?” or, alternatively, “How likely do you think it is that you will live beyond the age of 80?”. Such questions require minimal time, can be easily integrated into lifestyle counseling, and provide valuable insight into a patient’s future-oriented motivation. A low SLE may indicate reduced engagement in long-term health investments and identify individuals who require additional motivational support. In practice, SLE can be used to personalize lifestyle recommendations. For example, individuals reporting low SLE may benefit from interventions emphasizing short-term, tangible gains of improved sleep and PA (e.g., better energy, mood, and daily functioning), whereas those with higher SLE may be more receptive to long-term preventive framing focused on healthy longevity. Similarly, nutritional counseling can be adapted by linking dietary improvements either to immediate functional benefits or to future health preservation, depending on the individual’s longevity expectations. In this way, SLE functions as a clinically useful psychological marker that can guide the tailoring of sleep, PA, and nutritional recommendations within a personalized preventive framework.

## 5. Conclusions

This narrative review integrates evidence on how SQ, PA, and psychological determinants form the core system shaping both biological and perceived aging. Sleep and PA act as primary behavioral drivers of cardiometabolic, neurocognitive, inflammatory, and autonomic processes underlying healthspan and longevity, while dietary behavior and nutritional status function as clinically relevant biological modulators of these mechanisms. Psychological factors—including health perception, emotional resilience, optimism, perceived control, and SLE—regulate motivation, self-regulation and engagement in long-term health-promoting behaviors.

A key contribution of this review is the proposed conceptual model positioning SLE as a central psychological mechanism linking lifestyle behaviors to long-term motivation, self-regulation, and health outcomes. Although sleep, PA and dietary habits provide the biological foundation for healthy aging, SLE and related psychological resources act as higher-order regulators that amplify or attenuate the effects of these behaviors. Higher SLE is associated with sustained engagement in health-promoting routines, whereas lower SLE may contribute to behavioral disengagement and accelerated subjective and biological aging, helping to explain heterogeneous aging trajectories among individuals with similar clinical profiles.

The proposed model conceptualizes aging as a dynamic, bidirectional system that is intended as a hypothesis-generating framework, in which sleep influences psychological functioning, psychological functioning shapes perceived longevity, and perceived longevity guides health behavior choices that feed back into sleep and physical health, within a broader lifestyle context supported by nutritional status and dietary quality. This systems-based perspective supports the need for integrative interventions targeting sleep hygiene, circadian stability, PA, stress regulation, nutrition and longevity-related cognitions simultaneously, as such approaches may yield synergistic benefits beyond isolated behavior change strategies.

The findings further highlight the importance of contextual moderators—including age, gender, socioeconomic status, cultural beliefs, and engagement with wearable technologies—in shaping responses to lifestyle interventions, underscoring the need for tailored and equitable strategies. At the same time, major gaps remain, including the need for longitudinal and experimental studies to clarify causal directionality, standardized assessment of SQ, and multidimensional biomarkers integrating behavioral, biological, nutritional and psychological domains. Interventions explicitly targeting SLE represent a promising yet largely unexplored avenue for enhancing long-term adherence to healthy lifestyles.

In summary, healthy aging reflects the convergence of biological processes, daily lifestyle behaviors, and psychological expectations about the future. Strengthening these interconnected pathways may enable clinicians, researchers, and policymakers to promote not only longer lives, but also more positive and meaningful perceptions of aging as a modifiable trajectory shaped by behavior, biology, and belief.

## Figures and Tables

**Table 1:** Summary of ten key studies examining relationships among sleep, PA, psychological factors, autonomic function, and aging. The table highlights how each study informs one or more components of the integrative model proposed in this review.

Study (Author, Year)	Design & Sample	Key Variables	Main Findings	Relevance to Integrative Model
1. Panagiotou et al., 2021 [13] The Aging Brain: Sleep, the Circadian Clock and Exercise	Mechanistic narrative review	Sleep architecture, circadian rhythms, neuroinflammation, exercise	Aging reduces circadian amplitude and SWS; exercise enhances neuroplasticity (BDNF) and improves sleep	Strong mechanistic support for Sleep ↔ PA ↔ Circadian Stability
2. Barragán et al., 2021 [14] Physical Activity, Smoking, Sleep and Telomere Length	Systematic review (83 studies)	Telomere length, sleep duration/quality, PA, cellular aging	Poor sleep and inactivity predict shorter telomeres; PA and good sleep linked to slower biological aging	Supports biological aging pathways (oxidative stress, inflammation)
3. Shi et al., 2024 [11] Sedentary Behavior, Light Activity and Healthy Aging	Large JAMA cohort study	Sedentary time, light PA, healthy aging index	Light PA and less sedentary time strongly associated with healthier aging trajectories	Reinforces PA → healthspan, importance of movement across the day
4. Yao et al., 2025 [15] 24-h Movement, Mental Health & Cognition	Review	Sleep, PA, sedentary behavior, mental health, cognition	Integrated 24 h movement patterns strongly influence mental health and cognition; synergistic interactions	Aligns with holistic daily behavior framework central to integrative model
5. Sato et al., 2021 [16] Autonomic Nervous Activity, Exercise, and Sleep in Older Adults	Narrative review	HRV, autonomic regulation, sleep efficiency, exercise	Exercise improves HRV; higher HRV connected to better sleep and functioning	Provides autonomic mechanism linking PA ↔ Sleep ↔ Aging
6. Christie et al., 2016 [17] PA, Sleep Quality and Fatigue	Observational; adults across lifespan	PA, subjective SQ, fatigue	PA predicts better SQ and lower fatigue; strongest associations in older adults	Supports behavioral cascade PA → Sleep → Daytime vitality
7. Vanderlinden et al., 2020 [10] PA Programs and Sleep in Older Adults	Systematic review of interventions	Structured PA, sleep outcomes (duration, efficiency)	Controlled PA programs improve sleep; real-world programs show inconsistent sleep benefits	Highlights contextual moderators (setting, adherence, sleep baseline)
8. Lee et al., 2025 [18] Daily Reciprocal Relationships Between Affect, PA, and Sleep	Daily diary + accelerometry	Affect, PA, SQ/duration	Positive affect increases sleep & PA; poor sleep reduces next-day PA; strong day-level reciprocity	Demonstrates affect-driven feedback loops central to model
9. Williams et al., 2021 [19] 24-h Movement Spectrum & Vascular Remodeling	Narrative review	Sedentary behavior, light/moderate/vigorous PA, vascular aging	PA across intensities improves vascular remodeling; sedentary time accelerates vascular aging	Adds vascular aging mechanism to PA pathways toward longevity
10. Hakimi et al., 2023 [20] PA, Sedentary Behavior, Sleep and QoL in Older Adults	Systematic review	PA, sedentary behavior, sleep duration, QoL	Higher PA and adequate sleep predict better quality of life; sedentary behavior harms perceived health	Links behavioral patterns → QoL → subjective aging, supporting role of psychological pathways

Abbreviations: PA—physical activity; HRV—heart rate variability; QoL—quality of life. The ten studies included in this summary were selected based on conceptual relevance to the domains of sleep, PA, autonomic and psychological functioning, and aging-related outcomes. The table is intended as a representative synthesis, not an exhaustive systematic mapping of the literature and the studies listed were selected to represent key biological, behavioral, and psychological pathways relevant to the proposed model.

## Data Availability

No new data were created or analyzed in this study. Data sharing is not applicable to this article.
